# ﻿Morphology and molecular phylogeny of *Monomicrocaryon trimarginalis* sp. nov. (Ciliophora, Hypotricha), a new soil ciliate species from South Korea

**DOI:** 10.3897/zookeys.1258.168452

**Published:** 2025-11-05

**Authors:** Kyu-Seok Chae, Gi-Sik Min, Kyung-Min Park

**Affiliations:** 1 Department of Biological Sciences and Bioengineering, Inha University, Incheon 22212, Republic of Korea Inha University Incheon Republic of Korea; 2 Department of Biological Resources Research, National Institute of Biological Resources, Incheon 22689, Republic of Korea Department of Biological Resources Research, National Institute of Biological Resources Incheon Republic of Korea

**Keywords:** Ciliate, Oxytrichidae, SSU rRNA gene, terrestrial, taxonomy

## Abstract

The morphology and molecular phylogeny of the new soil ciliate, *Monomicrocaryon trimarginalis***sp. nov.**, which was discovered in the soil of from Gwangdeok Mountain, Hwacheon-Gun, South Korea, were investigated. The new species is characterized by the following morphological features: cell ellipsoidal to slightly ovate, with both ends rounded; cortical granules absent; 17–24 adoral membranelles; three frontal cirri; four frontoventral cirri, two postoral ventral cirri; one buccal cirrus; 10–13 left and 9–13 right marginal cirri; five transverse cirri; four dorsal kineties and three dorsomarginal kineties; two macronuclear nodules and one micronucleus in between macronuclear nodules. Phylogenetic analyses showed that *M. trimarginalis***sp. nov.** is placed within a clade containing *M. euglenivorum euglenivorum* and species belonging to *Quadristicha* in the dorsomarginalian hypotrichs.

## ﻿Introduction

Hypotrichia Stein, 1859 is one of the most well-known ciliate taxa and has been discovered in over 1,000 species across in soil, freshwater, brackish water, and seawater worldwide. Researchers have discovered many new Hypotrichia species, and the number is expected to exceed previous expectations (Berger and Foissner 1987, 1989, 1997; Berger 1999, 2001, 2006, 2008, 2011; Foissner et al. 2002, 2008; Foissner 2016; Kumar and Foissner 2016; Paiva 2020; Foissner and Berger 2021; Omar et al. 2024). Oxytrichidae Ehrenberg, 1838 is the largest family within Hypotrichia, and it is defined by important characteristics, such as the presence of 18 frontal-ventral-transverse cirri clustered into six recognizable groups, which usually arise from six longitudinal anlagen, dividing into a 1:3:3:3:4:4 cirral pattern (Berger 1999; Shao et al. 2015).

The genus *Monomicrocaryon* was divided into Oxytricha by Foissner (2016), who found that *Monomicrocaryon* has morphological features that differ from those of *Oxytricha*, mainly by one micronucleus between two macronuclear nodules. To date, 17 species of *Monomicrocaryon* have been identified: *M. granulatum* (type species), *M. alfredi*, *M. balladyna*, *M. crassicirratum*, *M. elegans*, *M. euglenivorum euglenivorum*, *M. euglenivorum fimbricirratum*, *M. geleii*, *M. halophilum*, *M. kahlovatum*, *M. longicirratum*, *M. opisthomuscorum*, *M. parahalophilum*, *M. pseudofurcatum*, *M. pseudofusiformis*, *M. saprobia*, and *M. sphagni* (Foissner 2016).

The present study describes a new soil ciliate discovered in the surface soil layer from Hwacheon-Gun in South Korea. Based on observations of its morphology using living cell and protargol impregnations and its position in the phylogenetic tree inferred from its 18S rDNA sequence, we classified the new species within the genus *Monomicrocaryon*.

## ﻿Material and methods

### ﻿Sample collection and light microscopy

*Monomicrocaryon trimarginalis* sp. nov. was collected from the topsoil layer (0–5 cm) at Gwangdeok Mountain, Hwacheon-Gun, South Korea (38°06'44.9"N, 127°25'57.4"E) in May 2023. The sampling site is situated within a forest. The forest floor was densely covered with leaf litter and providing a moist, organic-rich microhabitat favorable for soil ciliates. The soil at the site was visually identified as clay-rich, with a dense and compact texture. The soil temperature at the time of sampling was approximately 21.1 °C, with a relative humidity of about 65.8%.

The soil sample was left to air-dry for two weeks before being rehydrated with mineral water (Dongwon Saemmul, Dongwon Dear Food Co., South Korea) to trigger the excystment of ciliates. This was performed using the non-flooded Petri dish method as described by Foissner et al. (2002). Observations of living specimens were conducted using a stereomicroscope (Olympus SZH10, Japan) and light microscopes (Leica DM2500 and Olympus BX53) equipped with differential interference contrast, at magnifications ranging from 50 to 1000×. The infraciliature was visualized through protargol impregnation, with the protargol powder being synthesized according to the method of Kim and Jung (2017) method, and the impregnation procedure following “Method A” from Foissner (2014). The general terminology was based on Lynn (2008), whereas specific terms related to hypotrichs follow Berger (1999, 2008) and Foissner (2016).

### ﻿Scanning electron microscopy

The scanning electron microscope technique was performed following the protocols outlined by Foissner (2014) and Moon et al. (2020). Briefly, the cells were fixed using a mixture of saturated aqueous mercuric chloride and 4% osmium tetroxide in a 1:1 ratio. Subsequently, the cells were washed multiple times with distilled water and mounted on glass coverslips coated with poly-L-lysine. Samples were processed using through an ethanol series and dried using a Quorum E3000 dryer (Quorum Technologies, East Sussex, UK). Finally, the specimens were coated and examined under a field emission scanning electron microscope (Hitachi S-4300, Tokyo, Japan).

### ﻿DNA extraction, PCR amplification, and sequencing

*Monomicrocaryon trimarginalis* sp. nov. was isolated from the raw culture using a glass Pasteur pipette under a stereomicroscope. The cells were rinsed at least five times with sterile distilled water and subsequently transferred to 1.5 ml centrifuge tubes, each containing a minimal volume of water. Genomic DNA was extracted following the protocol provided in the RED-Extract-N-Amp Tissue PCR Kit (Sigma, St. Louis, MO). For PCR amplification of the nearly complete SSU rRNA gene, the forward primer New EukA (5’-CTG GTT GAT YCT GCC AGT-3’) modified from Medlin et al. (1988) and the reverse primer LSU rev3 (5’-GCA TAG TTC ACC ATC TTT CG-3’) from Sonnenberg et al. (2007) were used. The optimized PCR conditions were as follows: initial denaturation at 95 °C for 2.5 min, followed by 40 cycles of denaturation at 95 °C for 30 s, annealing at 55 °C for 30 s, and extension at 72 °C for 4 min; with a final extension at 72 °C for 5 min. PCR products were purified using the QIAquick® PCR Purification Kit (QIAGEN, Hilden, Germany). Sequencing was performed using three internal primers 18S-F810 (5’-GCC GGA ATA CAT TAG CAT GG-3’), 18S-R300 (5’-CAT GGT AGT CCA ATA CAC TAC-3’), and 18S-F1470 (5’-TCT GTG ATG CCC TTA GAT GTC-3’) on an ABI 3700 sequencer (Applied Biosystems, Foster City, CA, USA).

### ﻿Phylogenetic analyses

The 18S rDNA sequence of *Monomicrocaryon trimarginalis* sp. nov. was aligned with 82 spirotrich sequences retrieved from the NCBI database. Four urostylids, *Anteholosticha paramanca* (KF806443), *Bakuella subtropica* (KC631826), *Diaxonella pseudorubra* (GU942564), and *Urostyla grandis* (EF535731), were selected as the outgroup species. All sequences were aligned using BioEdit (Hall 1999). The best-fit model GTR + I (0.7090) + G (0.4500) was selected under the Akaike information criterion (AIC) using jModelTest 2.1.10 (Darriba et al. 2012). Maximum likelihood (ML) analysis was conducted using iqtree version 2.3.6 (Nguyen et al. 2015) with 1,000 bootstrap replicates. The best evolutionary substitution models were chosen for each molecular marker individually based on the BIC criterion, using the built-in ModelFinder program (Kalyaanamoorthy et al. 2017). MrBayes 3.1.7 was used to perform Bayesian inference (BI) analysis with Markov chain Monte Carlo (MCMC) for 1,000,000 generations with a sampling frequency of every 100 generations and a burn-in of 2500 trees. The ‘prset’ command was used to implement prior parameters in BI analyses (prior parameters based on AIC output parameters from jModelTest). Pairwise distances were calculated using Mega 11 (Tamura et al. 2021). The phylogenetic trees were visualized in FigTree v. 1.4.4 by Rambaut (2016).

## ﻿Results

### ﻿Taxonomy

**Class Spirotrichea Bütschli, 1889**

**Subclass Hypotrichia Stein, 1859**

**Order Sporadotrichida Fauré-Fremiet, 1961**

**Family Oxytrichidae Ehrenberg, 1838**

**Genus *Monomicrocaryon* Foissner, 2016**

#### ﻿ Monomicrocaryon trimarginalis sp. nov.

Taxon classificationAnimaliaCiliophoraHypotricha

08E20446-E18D-51DA-86BE-6E34B0BEECDF

https://zoobank.org/29271F18-00C8-46B5-8891-8AA8C768DFDE

**Figure 1.**
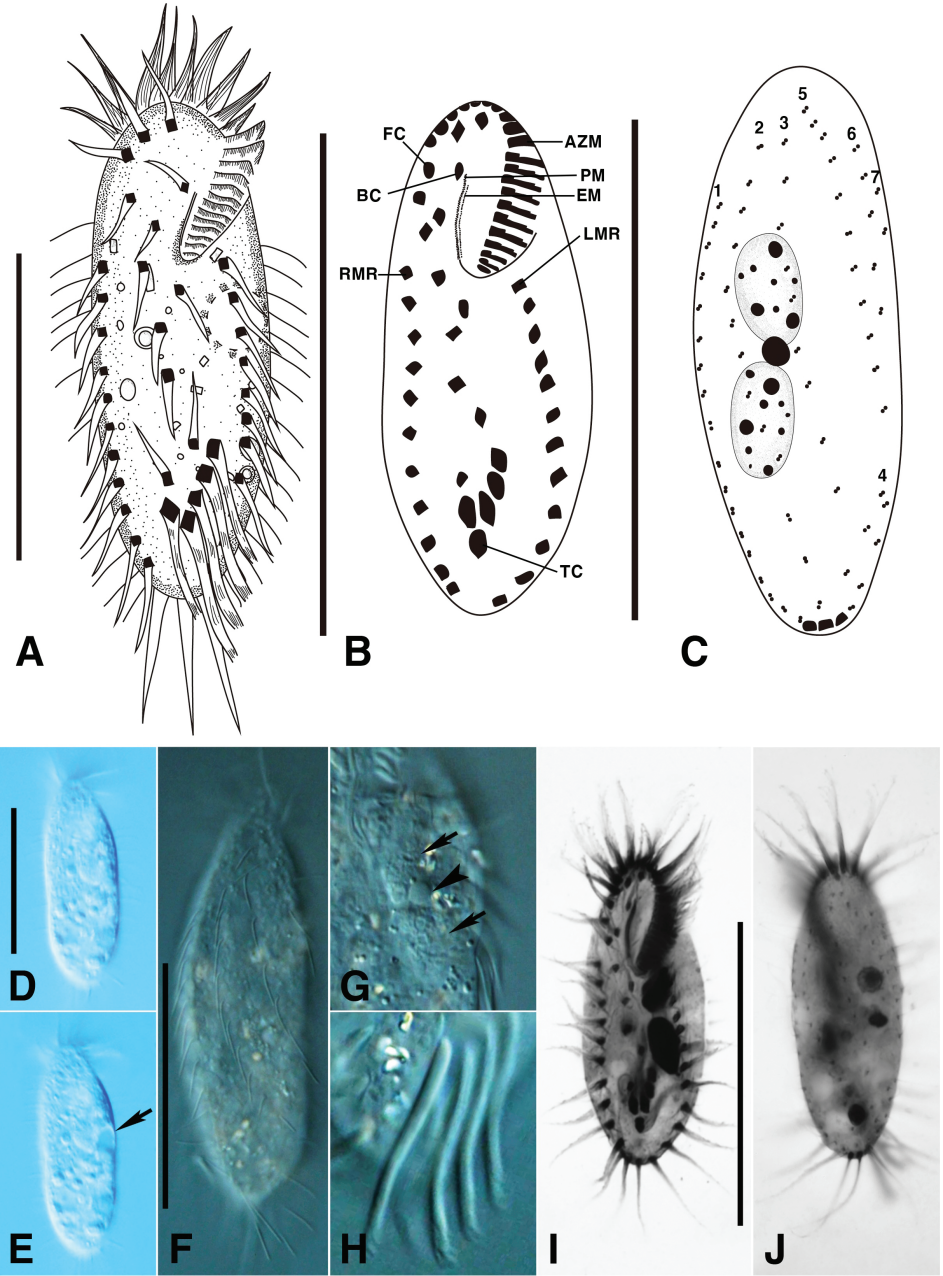
*Monomicrocaryon trimarginalis* sp. nov. from life (A, D–H), after protargol impregnation (B, C, I, J). A, Ventral view of a representative individual; B, C. Ventral (B) and dorsal (C) view of the holotype specimen; D, E. Ventral views showing the body shape and contractile vacuole (arrow); F. Dorsal view showing the dorsal bristles; G. Optical section showing the macronuclear nodules (arrows) and micronuclei (arrowheads); H. Ventral view showing the rod-shaped transverse cirri with fringed distal ends; I, J. Ventral (I) and dorsal (J) view of the holotype specimen. AZM, adoral zone of membranelles; BC, buccal cirri; EM, endoral membrane; FC, frontal cirri; LMR, left marginal row; PM, paroral membrane; RMR, right marginal row; TC, transverse cirri; 1–7, dorsal bristle rows. Scale bars: 50 μm (A–C, D, F, I).

**Figure 2.**
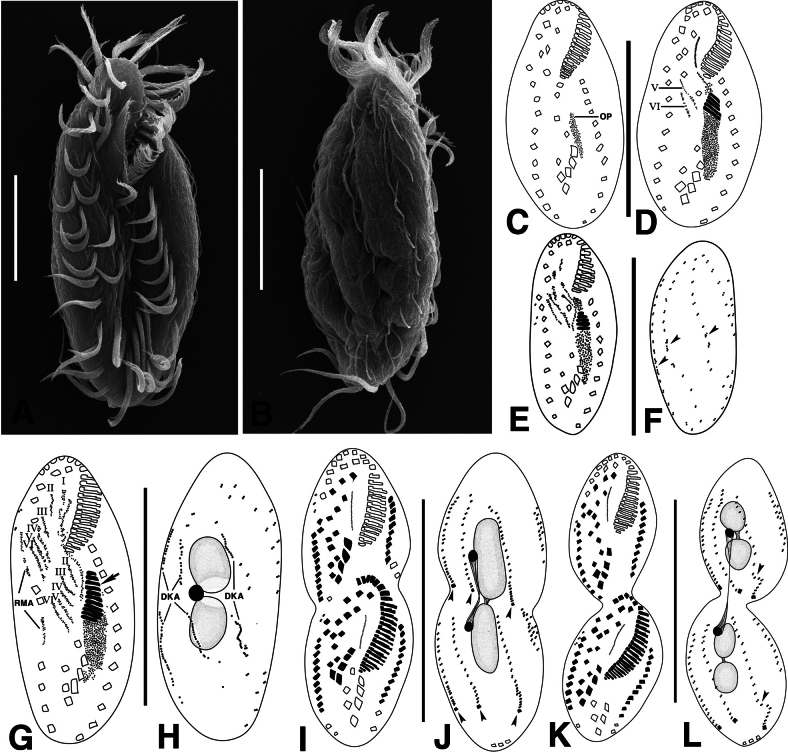
*Monomicrocaryon trimarginalis* sp. nov. under scanning electron microscope (A, B) and during morphogenesis (C–L) after protargol staining. A, B. Ventral and dorsal view showing the body shape and cirral pattern; C–H. Ventral and dorsal views of early dividers showing the formed the oral primordium, adoral membranelles (arrow), I–VI cirral anlagen and dorsal kineties anlagen (arrowheads); I, J. Ventral and dorsal view of a late divider showing caudal cirri (arrowheads); K, L. Ventral and dorsal view of a late divider showing fragmentation of the third dorsal kinety in each daughter cell (arrowheads). DKA, dorsal kineties anlagen; I–VI, frontoventral-transverse cirral anlagen 1–6; OP, oral primordium; RMA, right marginal anlage. Scale bars: 20 μm (A, B); 50 μm (C–L).

**Figure 3.**
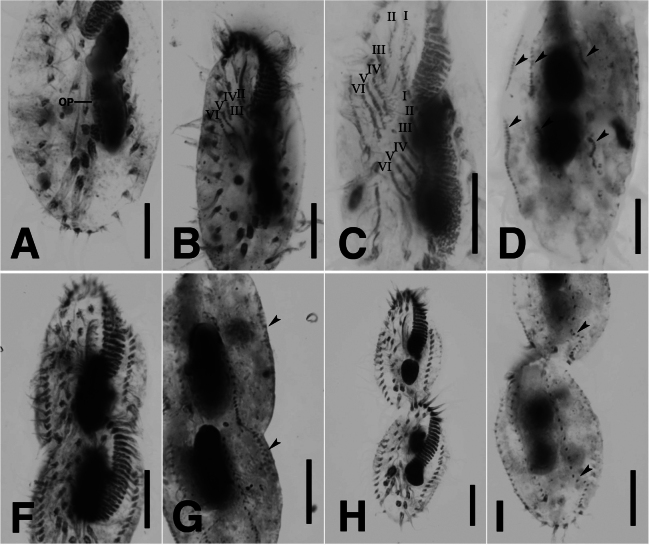
*Monomicrocaryon trimarginalis* sp. nov. during morphogenesis after protargol staining (A–I). A–D. Ventral and dorsal views of early dividers showing the formed the oral primordium, I–VI cirral anlagen, and dorsal kineties anlagen (arrowheads); F, G. Ventral and dorsal view of a late divider showing dorsomarginal kineties anlagen (arrowheads); H, I. Ventral and dorsal view of a late divider showing fragmentation of the third dorsal kinety in each daughter cell (arrowheads). I–VI, frontoventral-transverse cirral anlagen 1–6; OP, oral primordium. Scale bars: 10 μm (A–I).

Figs 1A–J, 2A–L, 3A–I, Table 1

##### Diagnosis.

Size 70–90 × 20–30 μm in vivo; body ellipsoidal to slightly ovate. Adoral zone occupies 40% of the body length and is composed of 17–24 membranelles. Two macronuclear nodules and one micronuclei between two macronuclear nodules. Cortical granules absent. Three frontal cirri; buccal cirrus; 9–13 right and 10–13 left marginal cirri; four frontoventral cirri; two postoral ventral cirri; five transverse cirri. Seven dorsal kineties; four dorsal kineties; and three dorsomarginal kineties.

##### Type locality.

Soil sample from Gwangdeok Mountain, Hwacheon-Gun, South Korea (38°06'44.9"N, 127°25'57.4"E) (for details, see Material and methods).

##### Type material.

Protargol-impregnated slide containing the holotype (Fig. 1B, C, I, J) (NIBRPR00001111990) and several paratype specimens (NIBRPR00001111991–NIBRPR00001111993) were deposited at the National Institute of Biological Resources (NIBR), South Korea. The holotype specimen was marked by a black ink circle.

##### Etymology.

Composite of the Latin prefix *tri*- and Latin adjective *marginalis*, referring to three marginal dorsomarginal rows.

##### Description.

Size 70–90 × 20–30 μm in vivo, and 39–54 × 16–25 μm (average 49 × 19 μm) in protargol preparations; length:width ratio about 2.5:1 (Table 1). Outline slightly ovate or elliptical, with both ends rounded (Figs 1A–F, I, J, 2A, B). Two globular to ellipsoidal macronuclear nodules, anterior nodule about 8 × 5 μm and posterior nodule about 9 × 5 μm in protargol preparations; one micronuclei, about 3 μm in diameter in protargol preparations, located between macronuclear nodules (Fig. 1C, G, I). Contractile vacuole slightly anterior to mid-body near left margin of the cell, approximately 10 μm in diameter when fully extended (Fig. 1A, D, E). Cytoplasm colorless, with lipid globules, many irregular crystals and food vacuoles.

**Table 1.** Morphometric data on *Monomicrocaryon trimarginalis* sp. nov.

Characteristic^a^	HT	Mean	M	SD	SE	CV	Min	Max	*n*
Body length	52	49.6	50.0	3.6	0.8	7.3	39	54	19
Body width	25	19.7	20.0	2.6	0.6	13.4	16	25	19
Body length:width, ratio	2.1	2.5	2.5	0.3	0.1	11.0	2.1	3.3	19
Anterior macronuclear nodule length	8	8.6	8.0	0.9	0.2	10.5	7	11	19
Anterior macronuclear nodule width	5	5.2	5.0	0.7	0.2	13.3	4	7	19
Posterior macronuclear nodule length	8	8.7	9.0	1.1	0.2	12.6	7	12	19
Posterior macronuclear nodule width	5	5.2	5.0	0.4	0.1	8.0	5	6	19
Macronuclear nodules, number	2	2	2	0	0	0	2	2	19
Micronucleus length	2	2.1	2.0	0.3	0.1	15.0	2	3	19
Micronucleus width	2	2.1	2.0	0.3	0.1	15.0	2	3	19
Micronucleus, number	1	1	1	0	0	0	1	1	19
Adoral zone length	21	19.7	20.0	1.3	0.3	6.7	18	22	19
Adoral zone membranelles, number	20	20.6	21.0	1.7	0.4	8.5	17	24	19
Body length: adoral zone length, ratio in %	40.4	39.9	40.4	3.3	0.7	8.2	34.0	46.2	19
Frontal cirri, number	3	3	3	0	0	0	3	3	19
Buccal cirri, number	1	1	1	0	0	0	1	1	19
Frontoventral cirri, number	4	4	4	0	0	0	4	4	19
Postoral ventral cirri, number	3	3	3	0	0	0	3	3	19
Pretransverse ventral cirri, number	2	2	2	0	0	0	2	2	19
Transverse cirri, number	5	5	5	0	0	0	5	5	19
Right marginal cirri, number	11	10.8	11.0	1.0	0.2	9.0	9	13	19
Left marginal cirri, number	11	11.2	11.0	1.0	0.2	9.1	10	13	19
Dorsal kineties, number	7	7.0	7.0	0	0	0	7.0	7.0	19
Kinetids in dorsal kinety 1, number	16	17.1	17.0	1.5	0.3	8.6	15	20	19
Kinetids in dorsal kinety 2, number	13	14.4	14.0	1.5	0.3	10.2	12	18	19
Kinetids in dorsal kinety 3, number	13	10.3	11.0	1.4	0.3	13.7	7	13	19
Kinetids in dorsal kinety 4, number	4	4.2	4.0	0.6	0.1	14.5	3	5	19
Kinetids in dorsal kinety 5, number	12	11.2	11.0	1.5	0.3	13.8	10	15	19
Kinetids in dorsal kinety 6, number	6	5.1	5.0	0.8	0.2	15.4	4	6	19
Kinetids in dorsal kinety 7, number	2	2.3	2.0	0.5	0.1	20.6	2	3	19

a Data based on randomly selected, protargol-stained specimens. All measurement in μm. CV, coefficient of variation in %; HT, holotype specimen; M, medium; Mean, arithmetic mean; Max, maximum; Min, minimum; *n*, number of specimens examined; SD, standard deviation; SE, standard error of the arithmetic mean.

Adoral zone occupies about 40% of body length and is composed of 17–24 membranelles, with cilia about 13 μm long (Fig. 1A, B, I; Table 1). Paroral and endoral membrane slightly bent and arranged in parallel (Fig. 1B, I). All cirri with cilia 8–15 μm long in vivo (Figs 1A, 2C). Eighteen frontoventral-transverse cirri: three frontal cirri close to the adoral zone of membranelles (Fig. 1A, B); buccal cirrus near the anterior end of undulating membranes. Three enlarged frontal cirri near the distal portion of the adoral zone, four frontoventral cirri, buccal cirrus near the anterior end of undulating membranes, and three postoral ventral cirri located in the central body, and two pretransverse cirri (Fig. 1B, I; Table 1). Five rod-shaped transverse cirri, about 25 μm long arranged in a V shape, each with a fringed distal end (Fig. 1B, H, I; Table 1). One left (10–13 cirri) and right (9–13 cirri) marginal row, both marginal rows non-confluent posteriorly (Fig. 1B, I; Table 1). Four dorsal and three dorsomarginal kineties with conspicuously long dorsal cilia 10–15 μm long. Dorsomarginal kinety 5 starts near the anterior end of the cell and extends to 2/3 of the body. Three caudal cirri located at the ends of dorsal kineties 1, 2, and 4 on the posterior end of the cell (Fig. 1C, J).

##### Morphogenesis.

Stomatogenesis begins with the development of a dense basal body that forms the oral primordium of the opisthe, which appears anterior to the leftmost transverse cirri (Figs 2C, 3A). A loosely arranged group of basal bodies develops anterior to the right side of the oral primordium, forming the undulating membrane anlagen of the opisthe (Fig. 2D, E).

In the opisthe, the scattered basal bodies at the anterior end of the oral primordium develop into frontoventral-transverse cirral anlagen (FVT anlagen) I, II and III of the opisthe (Fig. 2D). We were unable to determine whether FVT anlagen VI is formed from FVT anlagen VI of the proter or from IV/2. FVT anlagen V and VI originate from cirri V/4 and V/3, respectively (Fig. 2D). In the proter, FVT-anlagen II and III are newly formed from cirri II/2 and III/2, respectively (Figs 2E, G, 3B, C). FVT-anlagen IV develops from cirrus IV/3 (Fig. 2E, G). FVT anlagen V and VI originate from FVT anlagen V and VI of the opisthe (Figs 2G, 3C). Six frontoventral-transverse cirral anlagen are formed in both proter and opisthe (Fig. 3C). These cirral anlagen then broaden, break apart and migrate to their final positions as distinct cirri (Figs 2G, I, K, 3F, H). Cirri IV/2, VI/3 and VI/4 are not involved in the development of FVT cirri and are reabsorbed in later stages of division (Fig. 2I, K). The FVT anlagen I–VI form the pattern 1:3:3:3:3:4:4 in this order. (Figs 2I, K, 3F, H).

Right marginal anlagen develop within the right parental marginal row (Fig. 2G). Formation of left marginal anlagen by the basal body could not be observed. We confirmed that the development of the right marginal anlagen develops before the left marginal anlagen.

The dorsal ciliature is formed by two groups of primordia. The dorsal kineties anlagen develop intrakinetally within parental kineties 1–3 below the mid-body (Fig. 2F). Subsequently, these primordia proliferate, elongate, and divide to move into the proter and opisthe respectively (Figs 2H, 3D). In late dividers, the rightmost of the three ridges bends at the posterior end, giving rise to the formation of new dorsal kineties 3 and 4 (Figs 2J, L, 3I). Three caudal cirri are generated at the posterior end of each of the new dorsal kineties 1, 2 and 4 (Figs 2J, L, 3I).

##### Phylogenetic analyses.

**Figure 4.**
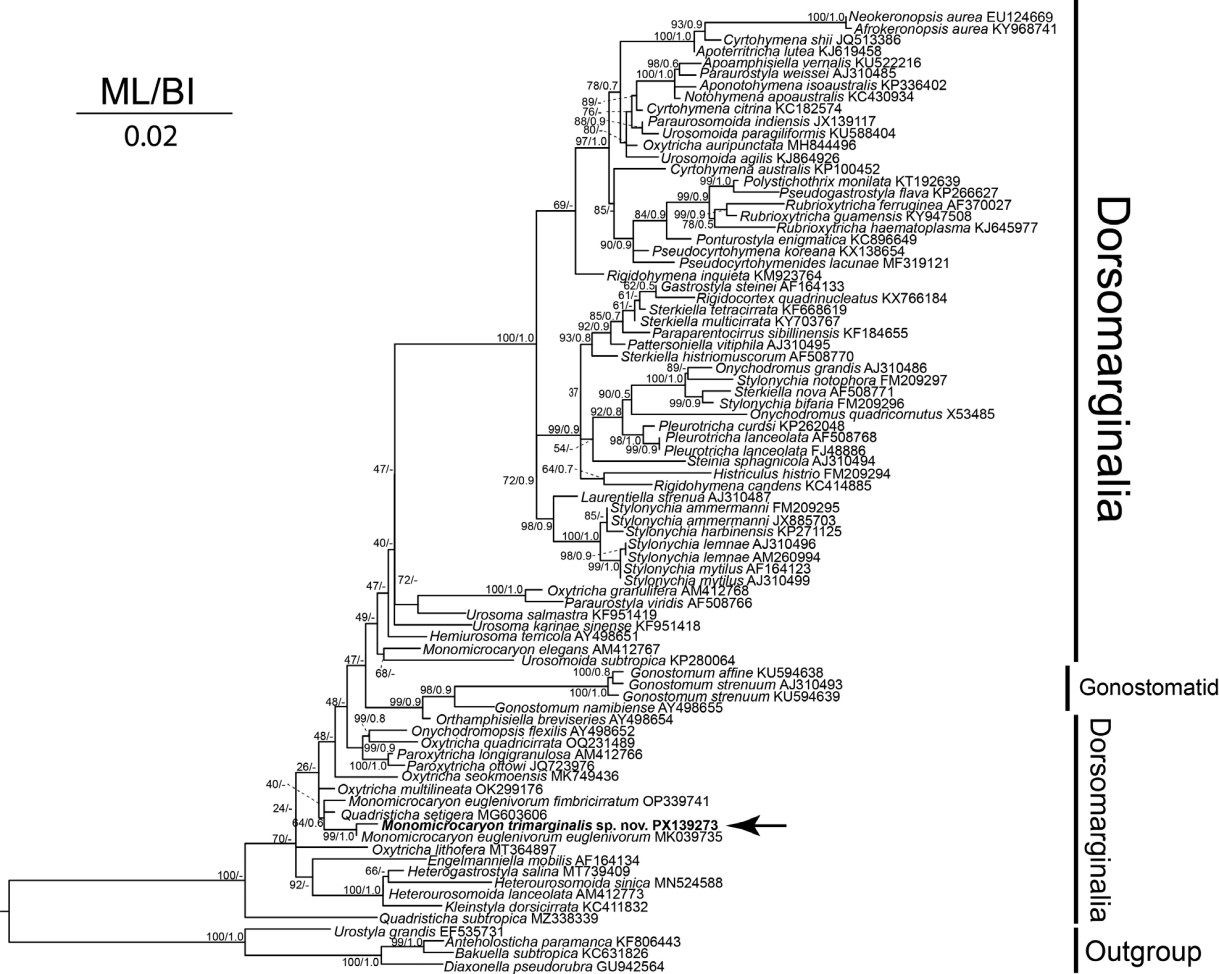
Maximum-likelihood (ML) phylogenetic tree based on 18S rRNA gene sequences, showing the position of *Monomicrocaryon trimarginalis* sp. nov. The new sequence is shown in bold and indicated by an arrow. The numbers at nodes indicate the ML bootstrap values and Bayesian-inference (BI) posterior probability. The estimated BI tree did not recover nodes designated with a dash (-). The scale bar corresponds to two substitutions per 100 nucleotide positions.

The SSU rRNA gene sequence of *Monomicrocaryon trimarginalis* sp. nov. (PX139273) has a length of 2909 bp and GC content of 46.5%. It differs from that of *M. euglenivorum fimbricirratum* (OP339741) in seven nucleotides, which corresponds to about 0.004 pairwise distance (Table 2). The pairwise distances and number of nucleotide differences between the available *Monomicrocaryon* species range from 0.004 to 0.012 and 7 to 20, respectively (Table 2). In the phylogenetic analysis, *M. trimarginalis* sp. nov. nests within a highly supported clade (99% ML / 1.0 BI) that also contains *M. euglenivorum euglenivorum*, and the species *M. euglenivorum euglenivorum*, and *M. trimarginalis* sp. nov., clusters with *Quadristicha setigera* with low support (64% ML/ 0.6 BI) (Fig. 4).

**Table 2.** Number of nucleotide differences (top right) and pairwise distances (bottom left) between SSU rRNA gene sequences of *Monomicrocaryon trimarginalis* sp. nov. and closely related species.

	1	2	3	4	5	6	7	8	9	10	11	12
1. *Monomicrocaryon trimarginalis* sp. nov.	–	3	6	7	12	20	20	25	28	33	36	37
2. *Quadristicha setigera*MG603606	0.002	–	8	8	13	20	19	26	30	36	37	41
3. *Oxytricha multilineata*OK299176	0.004	0.005	–	11	12	20	20	25	27	35	36	37
4. *Monomicrocaryon euglenivorum fimbricirratum*OP339741	0.004	0.005	0.007	–	17	22	23	27	30	36	38	42
5. *Monomicrocaryon euglenivorum euglenivorum*MK039735	0.007	0.008	0.007	0.010	–	26	24	29	30	39	41	35
6. *Oxytricha lithofera*MT364897	0.012	0.012	0.012	0.013	0.016	–	30	33	33	42	42	48
7. *Monomicrocaryon elegans*AM41276	0.012	0.012	0.012	0.014	0.015	0.018	–	35	39	41	46	41
8. *Heterourosomoida lanceolata*AM412773	0.015	0.016	0.015	0.016	0.018	0.020	0.021	–	5	12	17	54
9. *Heterogastrostyla salina*MT739409	0.017	0.018	0.016	0.018	0.018	0.020	0.024	0.003	–	17	18	56
10. *Kleinstyla dorsicirrata*KC411832	0.020	0.022	0.021	0.022	0.024	0.026	0.025	0.007	0.010	–	24	62
11. *Heterourosomoida sinica*MN524588	0.022	0.023	0.022	0.023	0.025	0.026	0.028	0.010	0.011	0.015	–	65
12. *Pseudouroleptus caudatus*DQ910904	0.023	0.025	0.023	0.026	0.021	0.029	0.025	0.033	0.034	0.038	0.040	–

## ﻿Discussion

### ﻿Comparison of *Monomicrocaryon trimarginalis* sp. nov. with congeners

With the inclusion of *M. trimarginalis* sp. nov., the genus *Monomicrocaryon* now contains 18 species. Based on the body size, nuclear apparatus, and morphometric data, the new species should be compared with the following *Monomicrocaryon* species, namely, *Monomicrocaryon granulatum* Foissner, 2016 (type species), *M. euglenivorum euglenivorum* (Kahl, 1932) Foissner, 2016, *M. euglenivorum fimbricirratum* Foissner, 2016, *M. crassicirratum* Foissner, 2016, and *M. opisthomuscorum* (Foissner et al., 1991) Foissner, 2016 (Table 3).

**Table 3.** Comparison of *Monomicrocaryon trimarginalis* sp. nov. with similar species.

Characteristic^a^	*Monomicrocaryon trimarginalis* sp. nov.	*M. granulatum*	*M. opisthomuscorum*	*M. euglenivorum euglenivorum*	*M. euglenivorum fimbricirratum*	*M. crassicirratum*
Body, length in vivo (μm)	70–90 × 20–30	60–90 × 20–30	55–80 × 20–40	60–120 × 25–45	50–70 × 20–30	100–150 × 32–60
Body length: width, ratio	2.5	2.7	-	-	2.1	2.6
Cortical granules	absent	Present	absent	absent	absent	absent
AM, number	17–24	18–23	18–22	17–20	16–19	23–28
Dorsal bristles, length in vivo (μm)	10–15	7–8	8–12	10-15	7–10	5–10
Cirri in LMR, number	10–13	15–20	11–15	8–12	9–11	13–19
Cirri in RMR, number	9–13	9–13	11–16	7–10	8–10	13–20
DK, number	7	6	5–6	5	5	6
Anterior Ma, length	7–11 × 4–7	8–13 × 5–8	9–17 × 5–9	12	7–11 × 6–8	13–19 × 10–14
Mi, length	2–3 × 2–3	2–3 × 2–2.8	2.5–4.5 × 2–4.5	3 in diameter	2–2.5 × 1.5–2	3–5 × 3–5
Habitat	Terrestrial	Terrestrial	Terrestrial	freshwater	Terrestrial	Terrestrial
Data source	This study	Foissner (2016)	Petz and Foissner (1997)	Shao et al. (2019)	Foissner (2016)	Foissner (2016)

a AM, adoral membranelles; DK, dorsal kineties; LMR, left marginal row; Ma, macronuclear nodules; Mi, micronuclei; RMR, right marginal row.

*Monomicrocaryon trimarginalis* sp. nov. is most similar to *M. euglenivorum euglenivorum*. However, they differ in the number of dorsomarginal kineties (3 vs 1), distal end of dorsal row 5 (terminates about slightly posterior to mid-body vs terminates about mid-body), number of bristles in dorsal rows 1 and 5 (15–20 and 10–15 vs 10–14 and 5–8), and habitat (terrestrial vs freshwater) (Shao et al. 2019).

*Monomicrocaryon euglenivorum fimbricirratum* differs from *M. trimarginalis* sp. nov. in the number of dorsomarginal kineties (1 vs 3), distal end of dorsal row 5 (terminates about slightly posterior to mid-body vs terminates about mid-body), number of bristles in dorsal rows 1, 2, and 5 (9–12, 8–11 and 7–9 vs 15–20, 12–18 and 10–15), and gap of dorsal row 1 (present vs absent) (Foissner 2016).

*Monomicrocaryon granulatum* differs from *M. trimarginalis* sp. nov. in the cortical granule (present vs absent), number of left marginal cirri (15–20 vs 10–13), and number of dorsomarginal kineties (2 vs 3) (Foissner 2016).

*Monomicrocaryon opisthomuscorum* is distinguished from *M. trimarginalis* sp. nov. by the number of dorsomarginal kineties (2 vs 3), distal end of dorsal row 5 (terminates about mid-body vs terminates slightly posterior to mid-body), number of kinetids in dorsal kinety 6 (2–4 vs 4–6), and the hook-shaped anterior part of the buccal cavity (present vs absent) (Petz and Foissner 1997).

### ﻿Phylogenetic analyses

In the new phylogenetic tree, the genus *Monomicrocaryon* is polyphyletic, which is consistent with the result of previous studies (Shao et al. 2019; Zhang et al. 2022). In our analyses, the new species was clustered with *M. euglenivorum euglenivorum*, *M. euglenivorum fimbricirratum*, and *Quadristicha setigera*. The close relationship between these species is supported by several morphological features, including one micronucleus between two macronuclei, one right and one left marginal row, two pretransverse cirri, five transverse cirri, and three caudal cirri (Berger 1999; Foissner 2016; Shao at al. 2019). However, based on morphological character, *M. trimarginalis* sp. nov. can be easily distinguished from *Q. setigera* by the fragmentation of dorsal kinety 3 (present vs absent), and the numbers of dorso-marginal kineties (3 vs 1) (Berger 1999).

Although *M. trimarginalis* sp. nov. and *M. euglenivorum fimbricirratum* have similar morphological characteristics, they differ in the total number of dorsal kineties (7 vs 5) and gap of dorsal row 1 (present vs absent) (Foissner 2016). Thus, in the phylogenetic analysis, *M. trimarginalis* sp. nov. does not cluster with *M. euglenivorum fimbricirratum*.

## Supplementary Material

XML Treatment for Monomicrocaryon trimarginalis

## ﻿Additional information

### Use of AI

No use of AI was reported.

### Author ORCIDs

Kyu-Seok Chae https://orcid.org/0000-0002-9289-7059

Gi-Sik Min https://orcid.org/0000-0003-2739-3978

Kyung-Min Park https://orcid.org/0000-0002-6125-4405

### Data availability

All of the data that support the findings of this study are available in the main text.

## References

[B1] Berger H (1999) Monograph of the Oxytrichidae (Ciliophora, Hypotrichia). Monographiae Biologicae 78: 1–1080. 10.1007/978-94-011-4637-1_1

[B2] Berger H (2001) Catalogue of Ciliate Names 1. Hypotrichs. Verlag Helmut Berger, 1–206.

[B3] Berger H (2006) Urostyloidea, Monograph of the Urostyloidea (Ciliophora, Hypotricha). Monographiae Biologicae 85: 1–1301. 10.1007/1-4020-5273-1_1

[B4] Berger H (2008) Monograph of the Amphisiellidae and Trachelostylidae (Ciliophora, Hypotricha). Monographiae Biologicae 8: 1–737. 10.1007/978-1-4020-8917-6

[B5] Berger H (2011) Monograph of the Gonostomatidae and Kahliellidae (Ciliophora, Hypotricha). Monographiae Biologicae 90: 1–741. 10.1007/978-94-007-0455-8_1

[B6] Berger H Foissner W (1987) Morphology and biometry of some soil hypotrichs (Protozoa: Ciliophora). Zoologische Jahrbücher. Abteilung für Systematik 114: 193–239.

[B7] Berger H Foissner W (1989) Morphology and biometry of some soil hypotrichs (Protozoa: Ciliophora) from Europe and Japan. Bulletin of the British Museum, Natural History. Zoology 55: 19–46.

[B8] Berger H Foissner W (1997) Cladistic relationships and generic characterization of oxytrichid hypotrichs (Protozoa, Ciliophora). Archiv für Protistenkunde 148(1–2): 125–155. 10.1016/S0003-9365(97)80048-6

[B9] Darriba D Taboada GL Doallo R Posada D (2012) jModelTest 2: More models, new heuristics and parallel computing. Nature Methods 9(8): 772. 10.1038/nmeth.2109 PMC4594756 22847109

[B10] Foissner W (2014) An update of ‘basic light and scanning electronmicroscopic methods for taxonomic studies of ciliated protozoa’. International Journal of Systematic and Evolutionary Microbiology 64(1): 271–292. 10.1099/ijs.0.057893-0 24198058

[B11] Foissner W (2016) Terrestrial and semiterrestrial ciliates (Protozoa, Ciliophora) from Venezuela and Galápagos. Denisia 35: 1–912.

[B12] Foissner W Berger H (2021) Terrestrial ciliates (Protista, Ciliophora) from Australia and some other parts of the world. Series Monographiae Ciliophorae 5(1): 1–380.

[B13] Foissner W Agatha S Berger H (2002) Soil ciliates (Protozoa, Ciliophora) from Namibia (Southwest Africa), with emphasison two contrasting environments, the Etosha region and the Namib Desert. Denisia 5: 1–1459.

[B14] Foissner W Chao A Katz LA (2008) Diversity and geographic distribution of ciliates (Protista: Ciliophora). Biodiversity and Conservation 17(2): 345–363. 10.1007/s10531-007-9254-7

[B15] Hall T (1999) BioEdit: A user-friendly biological sequence alignment editor and analysis program for Windows 95/98/NT. Nucleic Acids Symposium Series 41: 95–98.

[B16] Kalyaanamoorthy S Minh BQ Wong TK Von Haeseler A Jermiin LS (2017) ModelFinder: Fast model selection for accurate phylogenetic estimates. Nature Methods 14(6): 587–589. 10.1038/nmeth.4285 28481363 PMC5453245

[B17] Kim JH Jung JH (2017) Cytological staining of protozoa: a case study on the impregnation of hypotrichs (Ciliophora: Spirotrichea) using laboratory-synthesized protargol. Animal Cells and Systems 21(6): 412–418. 10.1080/19768354.2017.1376707

[B18] Kumar S Foissner W (2016) High cryptic soil ciliate (Ciliophora, Hypotrichida) diversity in Australia. European Journal of Protistology 53: 61–95. 10.1016/j.ejop.2015.10.001 26844781

[B19] Lynn DH (2008) The ciliated protozoa: Characterization, classification, and guide to the literature. Springer, New York. 10.1007/978-1-4020-8239-9

[B20] Medlin L Elwood HJ Stickel S Sogin ML (1988) The characterization of enzymatically amplified eukaryotic 16S-like rRNA-coding regions. Gene 71(2): 491–499. 10.1016/0378-1119(88)90066-2 3224833

[B21] Moon JH Kim JH Quintela‐Alonso P Jung JH (2020) Morphology, morphogenesis, and molecular phylogeny of *Neobakuella aenigmatica* n. sp. (Ciliophora, Spirotrichea, Bakuellidae). The Journal of Eukaryotic Microbiology 67(1): 54–65. 10.1111/jeu.12753 31356708

[B22] Nguyen LT Schmidt HA von Haeseler A Minh BQ (2015) IQ-TREE: A fast and effective stochastic algorithm for estimating maximum-likelihood phylogenies. Molecular Biology and Evolution 32(1): 268–274. 10.1093/molbev/msu300 25371430 PMC4271533

[B23] Omar A Moon JH Jung JH (2024) Morphology and molecular phylogeny of two hypotrichous ciliates (Ciliophora, Spirotrichea) from South Korea, including *Hemiurosomoida koreana* n. sp. European Journal of Protistology 92: e126045. 10.1016/j.ejop.2023.126045 38100885

[B24] Paiva Tda S (2020) Systematic redefinition of the Hypotricha (Alveolata, Ciliophora) based on combined analyses of morphological and molecular characters. Protist 171(4): 125755. 10.1016/j.protis.2020.125755 32858402

[B25] Petz W Foissner W (1997) Morphology and infraciliature of some soil ciliates (Protozoa, Ciliophora) from continental Antarctica, with notes on the morphogenesis of *Sterkiella histriomuscorum*. The Polar Record 33(187): 307–326. 10.1017/S0032247400025407

[B26] Rambaut A (2016) FigTree 1.4.3. http://tree.bio.ed.ac.uk/software/figtree

[B27] Shao C Lu X Ma H (2015) A general overview of the typical 18 frontal-ventral-transverse cirri Oxytrichidae*s. l.* genera(Ciliophora, Hypotrichia). Journal of Ocean University of China 14(3): 1–15. 10.1007/s11802-015-2482-7

[B28] Shao C Hu C Fan Y Warren A Lin X (2019) Morphology, morphogenesis and molecular phylogeny of a freshwater ciliate, *Monomicrocaryon euglenivorum euglenivorum* (Ciliophora, Oxytrichidae). European Journal of Protistology 68: 25–36. 10.1016/j.ejop.2019.01.001 30677704

[B29] Sonnenberg R Nolte AW Tautz D (2007) An evaluation of LSU rDNA D1-D2 sequences for their use in species identification. Frontiers in Zoology 4(1): 6. 10.1186/1742-9994-4-6 17306026 PMC1805435

[B30] Tamura K Stecher G Kumar S (2021) MEGA11: Molecular evolutionary genetics analysis version 11. Molecular Biology and Evolution 38(7): 3022–3027. 10.1093/molbev/msab120 33892491 PMC8233496

[B31] Zhang T Wang J Lyu Z Wang Y Al-Rasheid KAS Shao C (2022) Morphology, morphogenesis and phylogeny of a new soil ciliate, *Bistichella sinensis* n. sp., and morphology of two oxytrichids (Ciliophora, Hypotrichia). European Journal of Protistology 86: e125934. 10.1016/j.ejop.2022.125934 36283149

